# Phase 1 durability study of a single dose of the live-attenuated cholera vaccine PanChol and the safety and immunogenicity of a second dose

**DOI:** 10.64898/2026.10.13.26365415

**Published:** 2026-10-14

**Authors:** Deborah R. Leitner, Stephen R. Walsh, Amy C. Sherman, Alisse Hannaford, Sudhir Doranga, Masataka Suzuki, George I. Balazs, Elliot Hammerness, Kaylee T. Nguyen, Natalie E Izaguirre, Akash R. Sinha, Mariana I. Haugh-Klockner, Alexandra Tong, Xiaofang Li, Damien M. Slater, Nirajan Puri, Jason B. Harris, Steven Piantadosi, Lindsey R. Baden, Matthew K. Waldor

## Abstract

**Background:**

Killed whole-cell oral cholera vaccines require multiple doses and have reduced efficacy in young children, limiting their utility. PanChol is a single-dose, oral live-attenuated cholera vaccine engineered from a current seventh pandemic El Tor O1 *Vibrio cholerae* strain. PanChol was shown to be safe and immunogenic in a previous phase 1a trial. In participants from this first-in-human trial, we aimed to assess the durability of the initial PanChol dose and the safety and immunogenicity of a second dose.

**Methods:**

This open-label phase 1a booster study was conducted at Brigham and Women’s Hospital (Boston, MA). Eligible participants were healthy adults aged 18–55 years without a history of cholera or gastrointestinal disorders and had received a PanChol dose ranging from 10 ^5^ -10 ^10^ colony-forming units (CFU) within the previous 322-1111 days. All participants received a booster dose of 2×10 ^7^ CFU. The primary outcome was safety, including solicited, unsolicited, and serious adverse events following the second dose of PanChol. Secondary endpoints were the magnitude of pre- and post-vaccination serum vibriocidal titers to both Inaba and Ogawa *V. cholerae* , including seroconversion (4-fold rise in titers over baseline), and stool shedding of PanChol organisms using quantitative and qualitative stool cultures. Comparisons of the amount of vaccine shedding in stool following primary and secondary immunization were used to assess the intestinal mucosal immunity elicited by the initial PanChol dose. All outcomes were assessed in all participants. This trial is registered with ClinicalTrials.gov , NCT05657782 , and is complete.

**Findings:**

Between Aug 25, 2025, and Feb 20, 2026, 20 healthy adults who previously received a single oral dose of PanChol were enrolled to receive a booster dose of 2×10 ^7^ CFU. Eleven (55%) of 20 participants were male and the median age was 43·6 (IQR 30·2-49·5). Ten (50%) were White and 17 (85%) were not Hispanic or Latino. Nine (45%) of 20 boost recipients reported at least one solicited adverse event; 13 (93%) of 14 events were mild, and all were transient. The most common solicited adverse event was nausea, reported by 4 (20%) participants; all cases were mild. Most unsolicited adverse events were mild; one was grade 3 and was deemed unrelated to vaccination. Two unsolicited adverse events were deemed related to vaccination (mild gassy sensation and heartburn, both of which occurred on day 1 and resolved the same day). In most participants, vibriocidal antibody titers measured on the day of boosting exceeded baseline titers prior to the initial dose (17 (85%) for Inaba and 14 (70%) for Ogawa). Fifteen (75%) of 20 boost recipients seroconverted vibriocidal antibody titers to both serotypes. After the boost, 17 (85%) participants had no or self-limited vaccine shedding, compared with 10% after the first PanChol dose.

**Interpretation:**

A second oral dose of PanChol had a favorable safety profile and induced vibriocidal seroconversion to both serotypes in 75% of boost recipients. Intestinal colonization of PanChol following the boost serves as a proxy for measuring mucosal immunity targeting *V. cholerae* . The decrease in in the abundance of PanChol fecal shedding following the second dose, compared with the first, suggests that a single dose of PanChol is sufficient to confer durable protective immunity against a cholera challenge. These observations underscore the need for further clinical development of PanChol.

**Research in context:**

**Evidence before this study**

Vaccines are important tools for cholera control but current killed oral cholera vaccines (OCVs) require multiple doses for maximum efficacy and have limited efficacy in young children. In adults, protection mediated by killed OCVs is ∼55% 1 year after vaccination and lower in children. While estimates of the duration of protection afforded by natural infection vary, it is generally thought that immunity following infection is more durable across all age groups. PanChol is a live-attenuated cholera vaccine derived from a contemporary El Tor *V. cholerae* strain expressing both Inaba and Ogawa serotype antigens that was recently found to be safe and immunogenic in a first-in-human trial. We searched PubMed from database inception to September 1, 2026, for studies evaluating the durability of immunity to cholera following vaccination using the terms “duration of immunity to cholera vaccines”, yielding 11 non-review clinical trial articles. The search was limited to the English language. Previous challenge studies have not investigated the durability of protection from intestinal colonization and shedding beyond 6 months post-vaccination.

**Added value of this study**

A second dose of PanChol was administered 322-1111 days after participants had received an initial oral dose. The second dose of the vaccine was safe and well-tolerated, and 15 (75%) of 20 boost recipients seroconverted vibriocidal titers to both Inaba and Ogawa serotypes. Vaccine shedding was monitored to gauge PanChol intestinal replication. In striking contrast to primary vaccination, where 95% of participants shed the vaccine, only 30% of booster recipients shed PanChol and half of these individuals had self-limited shedding.

**Implications of all the available evidence**

A single dose of PanChol elicited mucosal immune responses that prevented *V. cholerae* intestinal colonization for up to 3 years post-vaccination. These observations suggest that a single dose of PanChol could limit *V. cholerae* transmission within communities by blocking *V. cholerae* intestinal colonization. PanChol’s favorable safety profile and stimulation of long-lived mucosal immunity support its continued development as a new single-dose agent for cholera control. Given these positive findings in adults in a non-endemic setting, future studies of the vaccine’s performance in cholera-endemic regions and in a variety of age groups are warranted. Further advancement of PanChol will require the development of a scalable manufacturing process to produce a heat-stable product that can be easily deployed in cholera-endemic areas.

